# In vitro studies on the dependence of drug deposition in dentin on drug concentration, contact time, and the physicochemical properties of the drugs

**DOI:** 10.1007/s00204-023-03573-6

**Published:** 2023-08-16

**Authors:** Miriam Klima, Volker Auwärter, Markus J. Altenburger, Merja A. Neukamm

**Affiliations:** 1 Institute of Forensic Medicine, Forensic Toxicology, Medical Center-University of Freiburg, Freiburg, Germany; 2 Faculty of Medicine, University of Freiburg, Freiburg, Germany; 3 Department of Operative Dentistry and Periodontology, Center for Dental Medicine, Medical Center-University of Freiburg, Freiburg, Germany; 4 Department of Laboratory Medicine and Toxicology, Labor Berlin-Charité Vivantes GmbH, Berlin, Germany

**Keywords:** Teeth, Dental hard tissues, Forensic toxicology, Illicit drugs, Alternative matrices, LC–MS/MS

## Abstract

The chemical analysis of dental hard tissues can provide information on previous drug use due to the deposition of drugs into this tissue. For the interpretation of analytical results in, e.g., postmortem toxicology or regarding archeological samples, the influence of drug dosing, consumption frequency, duration of intake and type of drug on analyte concentrations in teeth has to be characterized. To approximate these correlations, in vitro models were applied to investigate the time dependency of drug deposition via and against pulp pressure (perfusion studies) and the concentration dependency of drug deposition via oral cavity (incubation study) as well as the influence of de- and remineralization (pH cycling) on the incorporation of drugs in bovine dentin pellets. Some of the drugs of abuse most relevant in forensic case work (amphetamines, opiates, cocaine and benzoylecgonine) were applied. Concentrations in dentin samples were analyzed by liquid chromatography–tandem mass spectrometry (LC–MS/MS) after pulverization and extraction via ultrasonication with methanol. The studies showed that drug deposition in dentin likely depends on the physicochemical properties of the drug molecules as well as on the duration of contact with drugs via the blood stream and on drug concentrations present in the oral cavity. Higher drug concentrations in teeth can result from a more frequent or longer drug use. In addition, intake of higher doses or oral/inhalative consumption can also be expected to lead to higher drug concentrations. These findings can be helpful for the interpretation of postmortem cases.

## Introduction

Medical and illicit drugs can be deposited in teeth. Chemical–toxicological analysis of dental hard tissues for drugs might therefore facilitate a retrospective detection of drug intake. For the exploration of intoxications in postmortem toxicology teeth are basically a suitable alternative matrix to commonly used material as blood and urine (Skopp 2004; Rubin 2018). In lethal intoxication cases, knowledge about dosage, duration and type of applied drugs can be crucial for the identification of the cause of death. In the case of severely altered corpses or prolonged postmortem intervals, teeth and bones are usually the only remaining material for toxicological analysis. Some studies on the detection of drugs, drugs of abuse and metabolites in postmortem or extracted teeth are published (Cattaneo et al. 2003; Garcia-Algar et al. 2003; Pascual et al. 2003; Pellegrini et al. 2006; Schüssl et al. 2013; Zeren et al. 2013; Klima et al. 2016; Ottaviani et al. 2016; Cippitelli et al. 2017; Krais et al. 2017), but it has not yet been systematically investigated whether there is a correlation between the dose ingested, the frequency, duration and type of drug and the concentrations in teeth. Also, it has not yet been studied to what extent the manner of drug consumption (oral, intravenous) influences drug deposition in dental hard tissues.

Different routes of deposition of drugs in teeth are conceivable based on tooth structure and physiological processes in the oral cavity. The largest part of the tooth consists of dentin that is covered by enamel forming the clinical crown and cementum on the root. It can be assumed that drugs present in the blood are secreted from the blood vessels and deposited either in the pulp tissue, in the dentin or in the dentinal tubules. The physiological pulp pressure of about 20–30 mmHg (Hülsmann 2008; Hellwig et al. 2018) leads to a constant outward flow of dentin liquor through the dentinal tubules to the root surface. If dentin is exposed to the oral cavity due to gingival recession, periodontitis, caries, or iatrogenic causes the physiological perfusion pressure and the outward flow are likely to reduce the deposition of substances from the oral cavity into exposed dentin (Matthews and Vongsavan 1994).

Besides the deposition of drugs by the dentine liquor into the dentin, de- and remineralization processes in the dentin are a second way of uptake of substances especially into the outer layers of the dentin. After intake of food or acidic/sugared drinks, the pH value in the oral cavity decreases, this leads to the loss of calcium and phosphate from enamel and dentin. This process is called “demineralization” (Altenburger et al. 2012). The pH value increases over time due to the saliva buffer or after tooth brushing, and minerals from the saliva are reabsorbed into the tooth (remineralization) (Barbakow 1983; Larsen and Fejerskov 1989). During this process, drugs present in the oral cavity and saliva can be incorporated into the tooth as well (Conforti et al. 2000). This route of drug deposition might be predominant in orally consumed or inhaled drugs, where high concentrations of drugs can be expected in the oral fluid in connection with drug consumption (Drummer 2005).

The perfusion of dentin and the natural process of de- and remineralization in the oral cavity are described in some in vitro models. In an incubation study the diffusion of radiolabeled chemicals (alcohols, organic acids, etc.) through enamel into dentin was investigated (Haustein et al. 1994). A positive correlation was found between the concentrations in dentin and the lipophilicity of the chemicals. In models investigating the diffusion of resin monomers through dentin with applied pressure, the concentrations of the monomers in the solution before and after perfusion through dentin were compared (Gerzina and Hume 1995, 1996; Tak and Usumez 2015; Mahdhaoui et al. 2017; Putzeys et al. 2018 and many others). Under back/counter pressure the diffusion into the pulpal space was reduced (Gerzina and Hume 1996). In those perfusion studies, however, the perfusion flow was disregarded and the concentrations of deposited analytes in dentin were not determined. “pH cycling” is a method to simulate the changes of the pH value in the oral cavity and its effect on dental hard tissue via the de- and remineralization phases. In a proof-of-concept study, a model for the deposition of drugs into dentin by pH cycling (9 days, concentration of drug solution: 1 µg/mL) had been optimized (Klima et al. 2015). The obtained drug-positive dentin samples were used to validate a liquid chromatography-tandem mass spectrometry (LC–MS/MS) method for the detection of the applied drugs of abuse (Spinner et al. 2014).

Aim of this study was to simulate drug deposition in dentin using various in vitro models to investigate dependencies between the deposited amount and contact time as well as properties of the drugs. As model drugs, amphetamine, methamphetamine, 3,4-methylenedioxyamphetamine (MDA), 3,4-methylenedioxymethamphetamine (MDMA), 3,4-methylenedioxy-*N*-ethylamphetamine (MDEA), morphine, codeine, 6-acetylmorphine (6-MAM), cocaine and benzoylecgonine (BE) were used, which are the most relevant drugs of abuse in postmortem case work. Using a perfusion model, we investigated the correlation between duration of drug contact via blood flow (with applied pressure) and drug concentrations in dentin. Furthermore, we tested the hypothesis that drug diffusion from the oral cavity into dentin is reduced by pulpal hydrostatic pressure (counterperfusion model). With an incubation model, we investigated the relationship between drug concentrations in the oral cavity and drug concentrations in dentin. In addition, the influence of de- and remineralization of dentin on the deposition of drugs present in the oral cavity was examined by pH cycling.

## Materials and methods

### Chemicals and reference standards

All solvents and substances were at least of analytical or HPLC grade. Acetic acid, calcium chloride, calcium chloride dehydrate, and potassium hydroxide were purchased from Merck (Darmstadt, Germany). Ammonium formate solution (10 M) and methylenediphosphonic acid were obtained from Sigma-Aldrich (Steinheim, Germany), thymol from Applichem (Darmstadt, Germany), potassium dihydrogen phosphate from Calbiochem (San Francisco, USA), hydrochloric acid (HCl) from VWR International (Fontenay-sous-Bois, France), and Ampuwa^®^ from Fresenius Kabi (Bad Homburg, Germany). Deionized water was prepared with a cartridge deionizer from ELGA LabWater (Celle, Germany). 2-propanol, formic acid, HEPES, and potassium chloride were obtained from Carl-Roth GmbH (Karlsruhe, Germany). Methanol was purchased from Honeywell Riedel-de-Haёn (Seelze, Germany) and Technovit^®^ from Haraeus Kulzer (Wehrheim, Germany). 6-Acetylmorphine hydrochloride trihydrate, d,l-amphetamine hydrochloride, benzoylecgonine, cocaine hydrochloride, codeine hydrochloride, d,l-MDA hydrochloride, d,l-MDEA hydrochloride, d,l-MDMA-hydrochloride, d,l-methamphetamine hydrochloride, and morphine hydrochloride trihydrate were purchased from Lipomed (Weil am Rhein, Germany). 6-Acetylmorphine-D3, amphetamine, amphetamine-D5, benzoylecgonine-D3, cocaine-D3, codeine-D3, MDA, MDA-D5, MDEA, MDMA, methamphetamine, and morphine-D3 were purchased from Cerilliant (Round Rock, TX, USA). 6-Acetylmorphine, cocaine, codeine, MDEA-D5, MDMA-D5, methamphetamine-D5, and morphine were obtained from LGC Standards (Wesel, Germany).

### Solutions

*Demineralization solution (pH 5.0)* was prepared according to Buskes et al. containing 3 mM calcium chloride dihydrate, 3 mM potassium dihydrogen phosphate, 50 mM acetic acid, 6 µM methylenediphosphonic acid and traces of thymol in Ampuwa^®^ (Buskes et al. 1985). By adding adequate volumes of 5 M potassium hydroxide the pH was adjusted to 5.0.

*Demineralization solution (pH 4.8)* for the pH cycling was prepared according to ten Cate el al. containing 1.5 mM calcium chloride, 0.9 mM potassium dihydrogen phosphate and 50 mM acetic acid in Ampuwa^®^. By adding adequate volumes of 5 M potassium hydroxide, the pH was adjusted to 4.8 (ten Cate et al. 2006).

*Remineralization solution (pH 7.0)* for the pH cycling was prepared according to ten Cate et al. (2006), but twice as highly concentrated. The solution contained 3 mM calcium chloride, 1.8 mM potassium dihydrogen phosphate, 260 mM potassium chloride, and 40 mM HEPES in Ampuwa^®^. By adding adequate volumes of 5 mM potassium hydroxide, the pH was adjusted to 7.0. To yield drug-containing or drug-free remineralization solution, the remineralization solution was mixed either with the respective volume of drug solution or with deionized water (1/1, v/v).

For the *drug solution*, reference standards were used as solids, where the weighed-in quantity for salts was calculated with a corresponding factor. The required amounts of substances were dissolved in deionized water in a volumetric flask and stored at 2–8 °C. Before use, the drug solution was diluted with remineralization solution (1/1, v/v).

### Calibration standards and controls

The stock solution was prepared by mixing appropriate volumes of reference solutions followed by dilution with methanol to a final concentration of 1.0 µg/mL for all analytes. To prepare the working solutions (100 or 200 ng/mL, 10 or 20 ng/mL, and 1.0 or 2.0 ng/mL) the stock solution was diluted with methanol and adequate volumes of amphetamine standard solution were added to reach a concentration twice as high as the other analytes. The internal standard working solution was prepared by mixing appropriate volumes of the reference solutions of deuterated standards and dilution to a final concentration of 50 ng/mL. The calibration standards were prepared by adding adequate volumes of the working solutions and 10 µL of the internal standard working solution to 50 mg of dried and powdered blank dentin pellets prior to extraction. Quality control samples were prepared in the same manner. Values above the highest calibration standard or below the lower limit of quantitation (LLOQ) were extrapolated and indicated as approximate values.

### Preparation of blank dentin pellets

Bovine teeth incisors of the second dentition were collected from freshly slaughtered cattle. Teeth were stored in thymol solution (0.1%) at 2–8 °C until further processing. After the gum was removed by a scalpel, round dentin pellets (diameter 5.0 mm) were drilled out of the tooth root with a dental unit (KaVo, Biberach, Germany), a contra-angle hand piece (KaVo, Biberach, Germany) and a water-cooled trephine drill (Gebr. Brasseler, Lemgo, Germany). Dentin pellets were always taken from the upper part of the tooth root, because of the position-dependent differences in the number and diameter of the dentinal tubules (Mjör and Nordahl 1996; Schilke et al. 2000). After embedding the dentin pellets in cold-curing resin (Technovit 4071^®^), they were ground and polished under water cooling with a grinding machine (Struers, Rødovre/Copenhagen, Denmark) and sand paper (Struers, Ballerup, Denmark) from both sides to a thickness of 1.0–1.1 mm or 1.2–1.3 mm, respectively, checked by a digital micrometer (Mitutoyo, Tokyo, Japan). Until use, the pellets were stored in a moist plastic container at 2–8 °C. Before use, the pellets were separated from the resin and sonicated (Bandelin, Berlin, Germany) for 10 min to remove any remaining resin.

### Initial demineralization

To create initial carious-like lesions for the pH cycling and incubation study, dentin pellets were stored in demineralization solution (pH 5.0) (Buskes et al. 1985). Every 2–3 days, the pH value of the demineralization solution was tested, readjusted if necessary and the solution in the bowls was renewed. After 7 days, the initial demineralization was checked by transverse microradiography (TMR, Philips Industrial Electronics, Kassel, Germany) and a microscope (Carl Zeiss AG, Oberkochen, Germany) using two pellets. A mineral loss of at least 1500 vol%/μm showed sufficient demineralization. If this value was not reached, the samples were demineralized again and the TMR control was repeated after a few days. After sufficient demineralization (7–14 days), the samples were rinsed with deionized water and stored in a moist plastic container at 2–8 °C.

### Simulation of perfusion

#### General experimental setup

**Fig. 1:**
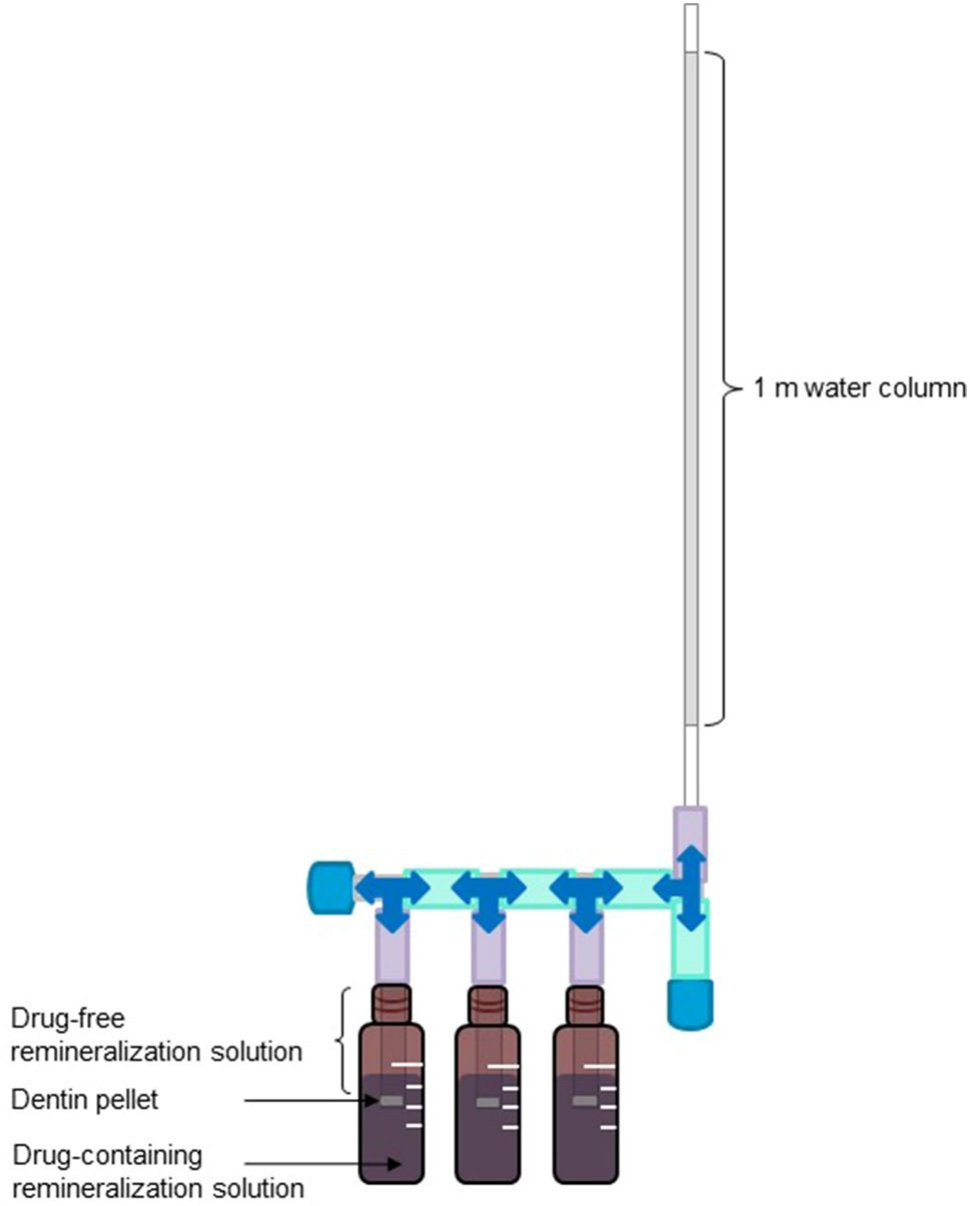
Experimental setup of the counterperfusion with drug-containing remineralization solution

The studies were conducted over a period of 5, 15, and 30 days. Dentin pellets with a thickness of 1.2–1.3 mm were used. Orthogonal exposure of the dentinal tubules was checked with the microscope and the dentin pellets were dried in the oven (Heraeus Holding GmbH, Hanau, Germany) at 37 °C for approximately 24 h. Six samples were applied per time period. Each pellet was fixed with the pulp side facing inward in a 3 cm tube section (Rauclair E 4 × 7 mm, Hans Kraeft GmbH, Hude, Germany) and connected via an adapter (B. Braun Melsungen AG, Melsungen, Germany) to a three-way valve (Discofix^®^, B. Braun Melsungen AG, Melsungen, Germany). Three samples were series connected and connected to a tube of approximately 120 cm length via another three-way valve. To simulate the physiological perfusion pressure (about 25 mmHg), a water column was used. The height of the water column in the tube was calculated using the following pressure conversions: 1 mmHg = 1.33 mbar; 1 mbar = 0.0102 mH_2_O. Accordingly, 25 mmHg corresponds to 0.34 mH_2_O. As three samples were connected in the experimental setup, and the calculated water column was multiplied by three (3 × 0.34 mH_2_O ≈ 1.0 mH_2_O). Therefore, 1 m of the tube was filled with water, and 20 cm of air was left in the lower part (see Fig. 1). To apply hydrostatic pressure on the samples, the valves were opened. For switching the solutions, the valves were closed shortly. The dentin was preconditioned with drug-free remineralization solution filled into the tube sections directly above the pellets and pressure was applied for 3 h. The bottom side of the pellets (facing outward in the tube) was also immersed in drug-free remineralization solution (filled into a bowl under the samples), representing the oral cavity.

#### Perfusion via pulp pressure

To simulate a drug consumption once daily and the contact of drugs via blood in the pulp, the solution in the tube sections directly above the pellets was replaced once a day for 3 h by a drug-containing remineralization solution (10 μg/mL). After 3 h, the tube sections as well as the samples were rinsed with drug-free remineralization solution. Then, the tube sections and the bowl under the samples were filled again with drug-free remineralization solution for about 21 h until next drug contact. The height of the water column was adjusted daily. After 5, 15 and 30 days, respectively, six samples were removed from the tube sections after the 21 h drug-free period. The samples were rinsed with deionized water, dried in the oven at 37 °C for approx. 24 h, and stored at − 20 °C until analysis.

#### Perfusion against pulp pressure (counterperfusion)

To simulate the deposition of drugs from the oral cavity against the physiological perfusion pressure into dentin, drug-containing remineralization solution (10 μg/mL) was filled into 4 ml glass vials and the dentin pellets clamped in the tubes were immersed in the solution for 3 h once a day (experimental setup see Fig. 1). During this time, the tube sections were filled with drug-free remineralization solution. After 3 h, the tube sections as well as the samples were rinsed with drug-free remineralization solution. Afterward, the tube sections and the bowl under the samples were filled with drug-free remineralization solution for 21 h until next drug contact. The height of the water column was adjusted daily. After 5, 15 and 30 days, respectively, six samples were removed from the tube sections after the 21 h drug-free period. The samples were rinsed with deionized water, dried in the oven at 37 °C for approx. 24 h, and stored at − 20 °C until analysis.

### Incubation in drug solution

As sample material, 27 initially demineralized dentin pellets were used. The samples were incubated in drug-containing remineralization solutions with three different concentrations (1 μg/mL, 3 μg/mL, and 6 μg/mL) and for three different times (1, 2, and 3 weeks). The pH value of the remineralization solution was tested every 2–3 days and readjusted if necessary. The remineralization solution was then diluted with appropriate volumes of drug solution and the sample solutions were renewed. After 1, 2 and 3 weeks, three samples each were rinsed with deionized water, dried in the oven at 37 °C for approx. 24 h and stored at − 20 °C until analysis.

### pH cycling

The deposition of drugs from the oral cavity into dentin during physiological changes of the pH value (demineralization and remineralization) was simulated for drug use four times a day with three different concentrations (0.2, 1.0, and 3.0 µg/mL) for 9 days. pH cycling was performed with remineralization and demineralization solution according to ten Cate el al. (2006). Before its use, the remineralization solution was diluted every day either with deionized water (night solution) or with drug solution to concentrations of 0.2 µg/mL, 1 µg/mL, and 3 µg/mL (day solutions). Three initially demineralized dentin pellets per concentration were used. The samples were placed for 30 min in demineralization solution and for 2.5 h in drug-containing remineralization solution repeated four times a day. The samples were stored overnight (12 h) in drug-free remineralization solution. After each change of the solution, the samples were rinsed with deionized water to avoid mixing the solutions. The pH value of the demineralization and remineralization solution was tested every 2–3 days and readjusted if necessary. After 9 days, the samples were rinsed with deionized water, dried in the oven at 37 °C for approx. 24 h, and stored at − 20 °C until analysis.

### Sample preparation

For pulverization, the dentin samples were placed separately (perfusion studies) or combined (pH cycling and incubation study) in the grinding bowl of a ball mill (MM400, Retsch, Haan, Germany) with a steel ball (diameter 10 mm) and ground for 1 min at a frequency of 20 Hz. Maximum 50 mg powder was transferred into micro cups and stored at − 20 °C. After adding 10 μL of the internal standard solution to the powder, extraction was performed three times with 500 µL methanol for 60 min in an ultrasonic bath (Bandelin electronic GmbH, Berlin, Germany). Then, the mixture was centrifuged for 10 min at 16,100×*g*. The supernatants were combined in a glass vial and evaporated at 40 °C under nitrogen flow to a small residual volume. To prevent evaporation of amphetamines, 100 μL 2-propanol/HCl (3/1, v/v) was added and the extract was evaporated to dryness. For the analysis, the residue was reconstituted in 100 μL of solvent A/B (95/5, v/v).

### Instrumental analysis

The samples were analyzed by LC–MS/MS using an API 5000™ Triple Quadrupole mass spectrometer (AB Sciex, Darmstadt, Germany) fitted with a Dionex UltiMate 3000 HPLC (Thermo Fisher, Dreieich, Germany). Chromatographic separation was achieved on a Luna PFP column (150 mm × 2 mm, 5 µm, Phenomenex, Aschaffenburg, Germany) with a guard column (4 mm × 2 mm, Phenomenex, Aschaffenburg, Germany) and gradient elution using solvent A (0.1% formic acid with 1.0 mmol/L ammonium formate in water) and solvent B (0.1% formic acid in methanol). The 15 min gradient started with 5% solvent B at 0.4 mL/min flow rate. After 1.0 min, solvent B increased up to 98% within 9.0 min at a flow rate of 0.6 mL/min and was kept for 2.0 min. Solvent B decreased to 5% within 0.5 min and the flow rate decreased to 0.4 mL/min within another 0.5 min. To re-equilibrate the system the initial conditions were kept for 2.0 min. A post-column addition of 2-propanol at a flow rate of 0.1 mL/min was applied to enhance sensitivity. Injection volume was 10 µL. The mass spectrometer was operated in positive ionization mode. Ion spray voltage was set to 2000 V. The gas settings were set as follows: curtain gas 40 psi, collision gas 9 psi, ion source gas (1) 60 psi, ion source gas (2) 70 psi. The ion source temperature was set to 400 °C. The multiple reaction monitoring (MRM) transitions and the corresponding MS parameters for all analytes and internal standards are shown in Table 1.

**Table 1:** MRM transitions, retention times, and corresponding voltages for the liquid chromatographic–mass spectrometric analysis of all analytes and internal standards

Analyte	Q1 [amu]	Q3 [amu]	DP [V]	EP [V]	CE [V]	CXP [V]	RT [min]
Amphetamine^a^	136.1	119.1	40	6.0	13	13	5.2
Amphetamine	136.1	91.0	40	6.0	25	14	5.2
Methamphetamine^a^	150.1	91.0	40	7.0	28	14	5.7
Methamphetamine	150.1	119.1	40	7.0	18	9.0	5.7
MDA^a^	180.1	135.1	40	6.0	32	15	5.5
MDA	180.1	105.1	45	6.0	31	16	5.5
MDMA^a^	194.1	163.2	40	6.0	20	13	5.9
MDMA	194.1	105.2	40	6.0	35	16	5.9
MDEA^a^	208.1	163.2	40	5.0	20	13	6.5
MDEA	208.1	105.1	40	5.0	37	16	6.5
Morphine^a^	286.2	152.0	130	9.0	78	17	3.3
Morphine	286.2	201.0	130	9.0	35	18	3.3
Codeine^a^	300.2	152.1	140	7.0	84	22	4.9
Codeine	300.2	115.2	140	7.0	94	19	4.9
6-Acetylmorphine^a^	328.1	165.1	150	8.0	54	20	5.2
6-Acetylmorphine	328.1	211.1	150	8.0	35	23	5.2
Cocaine^a^	304.2	182.3	40	9.0	28	14	7.1
Cocaine	304.2	82.0	40	9.0	45	15	7.1
Benzoylecgonine^a^	290.2	168.3	65	10	25	14	6.1
Benzoylecgonine	290.2	105.1	65	10	45	16	6.1
Internal standard:							
Amphetamine-D5	141.1	93.0	40	6.0	22	14	5.2
Methamphetamine-D5	155.1	92.0	40	7.0	28	14	5.7
MDA-D5	185.1	168.1	45	6.0	16	13	5.5
MDMA-D5	199.1	165.1	40	6.0	20	13	5.9
MDEA-D5	213.1	163.1	40	5.0	20	13	6.5
Morphine-D3	289.2	152.1	130	9.0	58	23	3.3
Codeine-D3	303.2	165.1	140	7.0	60	20	4.9
6-Acetylmorphine-D3	331.1	165.1	150	8.0	52	20	5.2
Cocaine-D3	307.2	185.1	40	9.0	28	14	7.1
Benzoylecgonine-D3	293.2	171.1	65	10	25	14	6.1

*Q1* mass-to-charge ratio of precursor, *Q3* mass-to-charge ratio of fragment, *amu* atomic mass unit, *DP* declustering potential, *EP* entrance potential, *CE* collision energy, *CXP* collision cell exit potential, *RT* retention time

^a^The transitions used for quantitation

### Statistical analysis

Statistical evaluation was performed for the perfusion studies. Differences between the groups (mean values after different time periods) were tested for significance at *p* < 0.05 level by *F*-Test (two-sample for variances) and *t*-Test (two-sample assuming unequal/equal variances) using Data Analysis in Microsoft Excel 2013.

## Results

### Perfusion via pulp pressure

Drug concentrations in dentin after 5, 15 and 30 days of perfusion with drug-containing remineralization solution (10 µg/mL) are shown in Table 2. Interestingly, cocaine showed the highest concentrations whereas the concentrations of BE were mostly lower than the concentrations of amphetamines (amphetamine, methamphetamine, MDA, MDMA, MDEA) and opiates (morphine, codeine, 6-MAM). There was about one order of magnitude between the concentrations of cocaine and BE after the respective perfusion period. After 15 days, the concentrations of each drug (except 6-MAM and BE) were two to three times higher than after 5 days (*p* < 0.05), but the concentration remained similar after 30 days (*p* > 0.05).

**Table 2:** Concentrations of model drugs in dentin pellets after different times of perfusion with drug-containing remineralization solution (10 µg/mL)

	5 days	15 days	30 days
Amphetamine	Approx. 5.3 ± 1.2	19 ± 4.6^c^	14 ± 3.7
Methamphetamine	3.9 ± 1.3	12 ± 3.5^b^	9.5 ± 2.3
MDA	8.3 ± 2.7	21 ± 5.5^c^	21 ± 4.6
MDMA	5.2 ± 1.9	15 ± 5.4^b^	14 ± 3.5
MDEA	4.9 ± 1.6	15 ± 6.0^b^	13 ± 4.5
Morphine	2.5 ± 2.3	8.7 ± 5.0^a^	9.4 ± 3.3
Codeine	2.2 ± 2.0	7.2 ± 3.9^a^	8.2 ± 4.1
6-MAM	2.7 ± 2.1	5.0 ± 2.0	(11 ± 4.5)
Cocaine	17 ± 4.1	37 ± 12^b^	43 ± 21
BE	1.2 ± 0.47	5.6 ± 4.4	3.8 ± 0.95

All values are the mean ± standard deviation of six experiments (except 6-MAM 30 days, *n* = 2)

^a^*p* < 0.05 compared to 5 days

^b^*p* < 0.01 compared to 5 days

^c^*p* < 0.001 compared to 5 days

### Perfusion against pulp pressure (counterperfusion)

Deposited drug concentrations in dentin after 5, 15 and 30 days of simulated contact in the oral cavity via diffusion against pulp pressure are shown in Table 3. The concentrations were generally about one third of those through regular perfusion (see Table 2). Cocaine showed the highest concentration difference between perfusion and counterperfusion with a factor of at least eight. Similar to the perfusion study, the lowest concentrations were found for BE after all three time periods, respectively. The concentrations of amphetamine remained almost constant throughout all periods studied (*p* > 0.05). For the other drugs, the mean concentrations in dentin increased significantly with increasing duration of counterperfusion (*p* < 0.05). After 5 and 15 days, the concentrations of opiates were relatively low compared to the other drugs. After 30 days, the concentrations increased six fold leading to the same concentration range as the other drugs (except BE), but the concentrations of opiates were highly scattered. Nevertheless, this effect cannot yet be explained and might be artefactual.

**Table 3:** Concentrations of model drugs in dentin pellets after different times of counterperfusion with drug-containing remineralization solution (10 µg/mL)

	5 days	15 days	30 days
Amphetamine	4.0 ± 1.0	4.6 ± 0.93	4.6 ± 1.8
Methamphetamine	1.7 ± 0.39	2.4 ± 0.69^a^	3.0 ± 1.3
MDA	3.3 ± 0.65	5.3 ± 1.6^a^	7.1 ± 2.1
MDMA	1.6 ± 0.33	2.9 ± 0.78^b^	5.1 ± 1.0^d^
MDEA	1.4 ± 0.34	2.3 ± 0.71^a^	5.3 ± 0.53^e^
Morphine	Approx. 0.49 ± 0.18	1.1 ± 0.45^a^	7.5 ± 4.7^c^
Codeine	0.44 ± 0.22	0.92 ± 0.39^a^	5.7 ± 3.5^c^
6-MAM	Approx. 1.1 ± 0.49	1.1 ± 0.49	6.2 ± 3.2^c^
Cocaine	2.4 ± 1.8	1.6 ± 0.60	5.2 ± 2.9^c^
BE	Approx. 0.17 ± 0.067	0.36 ± 0.095^b^	1.4 ± 0.50^d^

All values are the mean ± standard deviation of six experiments

^a^*p* < 0.05 compared to 5 days

^b^*p* < 0.01 compared to 5 days

^c^*p* < 0.05 compared to 15 days

^d^*p* < 0.01 compared to 15 days

^e^*p* < 0.001 compared to 15 days

### Incubation in drug solution

**Fig. 2:**
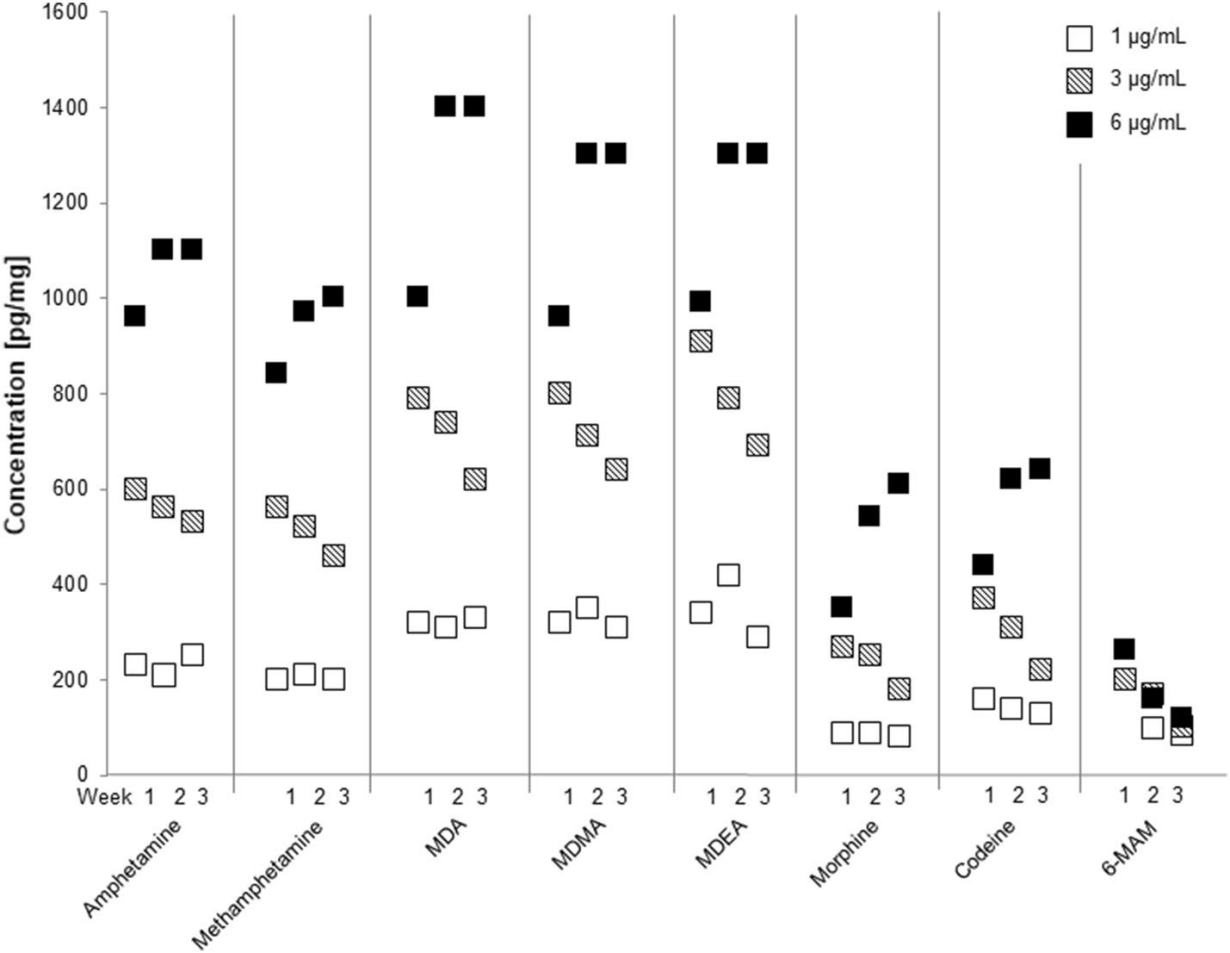
Mean concentrations of model drugs in demineralized dentin pellets (*n* = 3) after different times of incubation in drug-containing remineralization solution (1, 3 and 6 µg/mL). Due to analytical problems 6-MAM could not be analyzed after 1 week using the drug solution with a concentration of 1 µg/mL

**Fig. 3:**
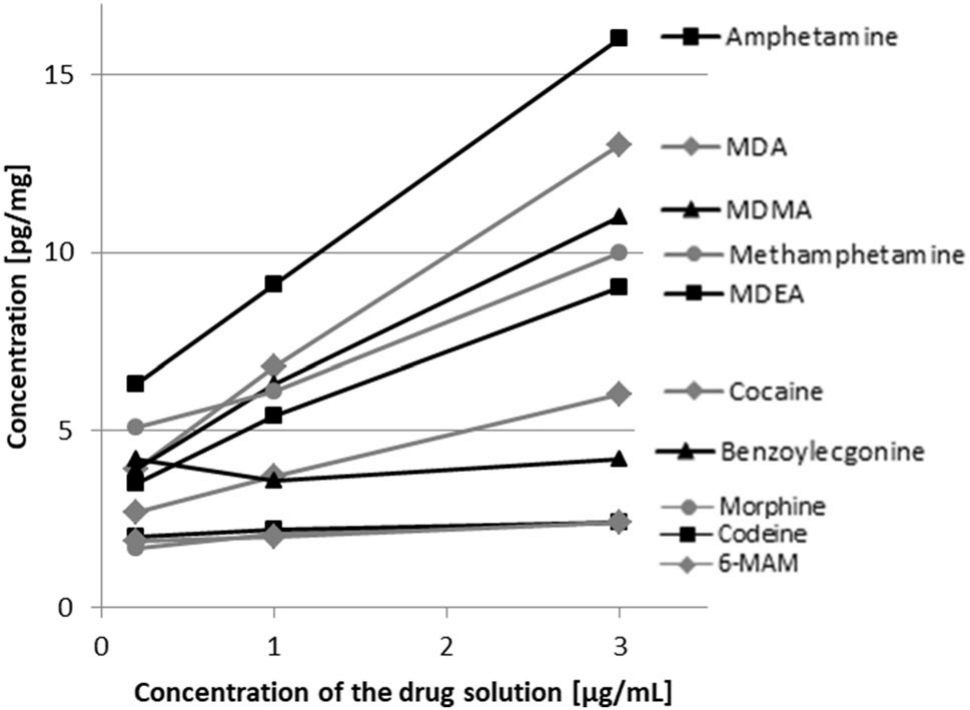
Mean concentrations of model drugs in demineralized dentin pellets (*n* = 3) after 9 days pH cycling with different concentrations of drug-containing remineralization solution

Drug concentrations in dentin after incubation of demineralized dentin pellets in drug-containing remineralization solution with concentrations of 1, 3 and 6 µg/mL for 1, 2, and 3 weeks are shown in Fig. 2. The mean concentrations in dentin after 1 week were higher than in the pH cycling study (see Fig. 3) by a factor of approx. 48 and 85 for the concentrations of 1 µg/mL and 3 µg/mL, respectively. The higher the concentration of the drug solution, the higher the drug concentrations in dentin. Moreover, this positive correlation seems to be linear. Incubation for 1, 2 or 3 weeks produced no clear differences in the drug concentrations in dentin. Concentrations of amphetamines were generally about two to three times higher than opiate concentrations. Cocaine and BE were not evaluated because of their instability under the incubation conditions (pH value 7.0).

### pH cycling

Drug concentrations in demineralized dentin after pH cycling with drug-containing remineralization solution (0.2, 1.0, and 3.0 µg/mL, four times a day) for 9 days are shown in Fig. 3. With increasing concentrations of the drug solution, concentrations of amphetamines and cocaine in dentin increased as well, whereas opiates and BE were very little to not affected from higher concentrations. Interestingly, despite a linear relationship, the increase in drug deposition in dentin is not proportional to the concentration of the applied drug solution (limit: only mean values available, no SD).

## Discussion

After 15 days of perfusion, the drug concentrations in dentin were two to three times higher than after 5 days, but there were similar concentrations after 15 and 30 days. With increasing duration of contact, an equilibrium between deposition and redissolution seems to be established. Drugs are incorporated against the physiological pulp pressure as well, but to a lower extent than via pulp perfusion. Since the concentrations of most drugs were much higher after 30 days than after 15 days, it can be assumed that the drug concentrations would still increase if the investigation period was extended. The concentration at which equilibrium between deposition and redissolution is established cannot be reached within 30 days of counterperfusion. Until the establishment of equilibrium, the deposition of drugs in dentin via and against pulp pressure seems to depend on the duration of drug contact. More frequent and longer-lasting drug use might therefore lead to higher drug concentrations present in authentic human dentin.

In contrast to the pH cycling and perfusion studies, the dentin samples in the incubation study were not in contact with drug-free solution at any time. Therefore, drugs could not redissolve from the samples and the drug concentrations in dentin mainly depend on establishment of diffusion equilibrium. As expected, the concentrations were substantially higher than in the pH cycling. In the incubation study, the deposition of drugs in dentin strongly correlates with the concentration of the applied drug solution and there seems to be a proportional relationship. In contrast, the duration of drug contact does not seem to play a role, as doubling or tripling the incubation time produced no distinct differences in the respective concentrations in dentin. Therefore, the deposition capacity of drugs in dentin might be limited. It seems that without redissolution phases, the concentration equilibrium is quickly reached (within 1 week).

In the pH cycling study, there is a visible correlation between applied concentrations of the drug-containing remineralization solution and deposition in dentin, which seems to be linear but not proportional. As the drugs show very different correlations to the applied concentration, there seem to be drug-related proportionality factors depending on their physicochemical properties. Nevertheless, it can be assumed that the general concentration of a drug in teeth depends on the drug concentration in the oral cavity. Therefore, higher drug doses as well as oral or inhaled drug intake are likely to lead to higher concentrations in teeth than low doses or parenteral drug application. The concentrations found in the presented study were much lower than in the study of Spinner et al., where the pellets were additionally stored overnight in drug-containing remineralization solution, leading to higher concentrations due to less redissolution phases (Spinner et al. 2014). Thus, duration of drug-free phases seem to play an important role for the concentrations in teeth as well.

Interestingly, the amount of the deposited drugs in dentin after incubation in drug solution differed between various drug/substance groups and seems to depend on the physicochemical properties of the drugs. Amphetamines are lower in molecular size and weight, and more hydrophilic than opiates, which might be the reason for the higher concentrations in the perfusion study. In contrast to Haustein et al. (1994), we found the small and hydrophilic amphetamines in higher concentrations than the more lipophilic opiates. This might be due to the alkaline nature of the drugs. The same distribution can also be seen less prominent in the pH cycling as well as in counterperfusion after 5 and 15 days. Opiates need a longer incubation period to reach higher concentrations in counterperfusion. Cocaine concentrations were at least eight times higher in perfusion than in counterperfusion, which could indicate that cocaine is deposited in dentin predominantly via blood flow of the pulp and less from the oral cavity. As the concentrations of all drugs except cocaine were mostly in the same concentration range in the perfusion study, the physicochemical properties of the drugs seem to play a minor role for deposition via the blood flow.

Limitations and perspectives of the presented studies were for example the use of dentin samples for in vitro studies, as variabilities in both diameter and density of the dentinal tubules running through dentin can lead to differences in the inner contact surface of the samples. For the best possible standardization, the dentin pellets were all made from the upper part of the tooth root, but still, the teeth were from a pool of different cattle. Therefore, larger concentration differences between the individual samples within an investigation group are to be expected, which was also evident in the relatively high standard deviations in the perfusion and counterperfusion studies (see Tables 2 and 3). The perfusion studies mainly simulated the deposition of drugs into root dentin. For future studies, the deposition in the crown of the tooth could be simulated as well using samples containing both enamel and dentin. Contact with drug-free solutions during pH cycling and perfusion studies obviously led to redissolution of deposited drugs. This effect can be used in future studies to differentiate permanent deposition of drugs from temporary/intermittent deposition with the presented models by extending the drug-free period at the end of the respective study. In vivo, from the oral cavity site, enamel, saliva/pellicle (Hannig et al. 2004) and the oral biofilm/plaque (Henkel et al. 2018, 2021; Roth et al. 2018) may influence drug diffusion into or deposition in dentin and therefore have an impact on establishment of equilibrium. This effect could be investigated using adaptions of the presented models.

## Conclusion

In this study, we simulated drug deposition of the most important drugs in forensic postmortem cases into dentin using different in vitro models to investigate dependencies between the deposited drug amount and the time/duration of contact as well as the physicochemical properties of the drugs. According to the perfusion model we can assume that drugs are incorporated into dentin via the blood stream. This incorporation should be proportional to the duration of regular drug use. However, after several days of regular drug use, concentration equilibrium seems to be reached.

Furthermore, diffusion into open dentinal tubules from the oral cavity (against pulp pressure) might contribute to the deposited drug amount in teeth. In the pH cycling model that takes the influence of de- and remineralization of dentin into account, it could be shown that higher drug concentrations in the oral cavity led to higher drug concentrations in dentin. In addition, the model drugs showed differences in their incorporation behavior. For the interpretation of authentic cases, it can be assumed that drug deposition in dentin depends on the physicochemical properties of the drug molecules, the duration of contact with drugs via the blood stream and on the drug concentrations present in the oral cavity. Higher concentrations in teeth can be the result of a more frequent or longer drug use. Other reasons for elevated drug concentrations could be the intake of higher doses or oral/inhalative consumption.

## Data Availability

The data supporting the findings of this study are available within the article or from the corresponding author, MAN, upon reasonable request.
